# Effect of Acupuncture on the Timeliness of Explosive Forces Generated by the Male Shoulder Joint

**DOI:** 10.1155/2021/5585605

**Published:** 2021-03-15

**Authors:** I-Lin Wang, Jun Wang, Yi-Ming Chen, Rui Hu, Yu Su, Shun Yao, Chun-Sheng Ho

**Affiliations:** ^1^College of Physical Education, Hubei Normal University, Huangshi 435002, Hubei, China; ^2^Jilin Sports University, No. 2476, Freedom Road, Nanguan District, Changchun, Jilin Provice 130022, China; ^3^Division of Physical Medicine and Rehabilitation, Lo-Hsu Medical Foundation, Inc., Lotung Poh-Ai Hospital, Yilan 26546, Taiwan; ^4^Department of Physical Therapy, College of Medical and Health Science, Asia University, Taichung 41354, Taiwan

## Abstract

Athletes aim to improve muscle strength to optimize sports performance and gain a competitive edge. Although modern sports medicine includes rehabilitation treatment methods for improving the explosive force of athletes, including acupuncture, a common alternative therapy, research on the effectiveness of acupuncture in improving the timeliness of explosive forces is limited. There is uncertainty regarding how long the effects of acupuncture treatment persist after treatment. Therefore, the purpose of this study was to explore the effect of acupuncture on the timeliness of explosive forces generated by the male shoulder joint. Eighteen healthy men underwent tests of shoulder adduction/abduction (Add/Abd) and flexion/extension (Flex/Ext) through an isokinetic measurement system. Acupuncture was used to stimulate LU1 (*Zhongfu*), LU3 (*Tianfu*), LU4 (*Xiabai*), LI14 (*Binao*), SJ13 (*Naohui*), SJ14 (*Jianliao*), and SJ12 (*Xiaoluo*), and the isokinetic parameters were recorded before and after acupuncture. After acupuncture, isokinetic muscle force parameters including the maximum (Max) torque, the average power, the average peak power, the average work, and the total work increased significantly (*P* < 0.05), whereas the average max torque Abd/Flex did not. Additionally, the preintervention values of the shoulder joints for Add/Abd and Flex/Ext were significantly greater than those at post 1 and post 2 (*P* < 0.05). The isokinetic results suggest that acupuncture can increase the explosive force of the male shoulder joint Add/Abd and Flex/Ext. Muscle cannot be fully activated when calcium saturation is below the maximal level. In this case, the postactivation potentiation (PAP) may enhance voluntary muscle force production. The effect of acupuncture is time-dependent, that is, the effects of acupuncture gradually weaken and disappear by approximately 10 minutes after acupuncture. Therefore, we suggest that acupuncture is used as an alternative therapy in sports competitions to increase the explosive forces of the shoulder joint, thereby improving sports performance.

## 1. Introduction

Explosive force is the ability of the muscles to increase the force generated rapidly from a small force or resting state [1]. Explosive force generation during athletic activities is often integrated with multidirectional movements involving high levels of neuromuscular activation [2].PAP is one of the influencing factors of explosive forces and represents an acute increase in muscle force velocity or explosive force caused by submaximal activation levels under short time of muscles reached prepotentiation [3]. The potential mechanism by which this event occurs is that PAP increases the excitability of *α*-motoneurons, resulting in an increase in their subsequent contractility performance after previous muscular activity [4]. The stimulation of nerves by acupuncture is also one of the ways that explosive muscle forces can be increased. By stimulating nerves, acupuncture can cause changes in the excitability of cortical motor neurons [5] and the recruitment of more motor units and/or increase the activation of already active motor units to higher frequencies [6]. Additionally, acupuncture-induced motoneuron recruitment may increase the fast-twitch fiber contribution and thus enhance performance in subsequent explosive activities [7, 8]. Acupuncture has long been used in the clinic to help individuals recover from muscle fatigue after exercise and can improve speed abilities and lower extremity explosive strength [9, 10]. A recent study showed that acupuncture at specific areas of the shoulder joint can increase muscle excitability, thereby delaying muscle fatigue and increasing muscle endurance [11]. Another study showed that fatigue resolution is associated with increased muscle strength, which is attributed to the PAP effect, which is caused by the increased excitability of *α*-motor neurons [12]. Acupuncture and PAP seem to have similar effects. After stimulation, they increase the excitability of motor neurons and thus enhance explosiveness and athletic performance. However, PAP stimuli also induce a state of fatigue, and individuals require a sufficient rest period before performing a subsequent explosive movement [13]. Therefore, when acupuncture is used to enhance muscle explosive forces, the effect on the timeliness of force generation should be studied.

Past studies have shown that PAP can lead to improvements in explosive sports performance [14]. A potential reason is that the excitation of the central nervous system caused by PAP stimulus leads to the increased effect of motor neuron recruitment and strength generation for 5 to 30 minutes [15]. Additionally, explosive performance is enhanced within 3–5 min after heavy resistance exercise (HRE) [16]. However, some research results show that the explosiveness does not immediately increase after HRE [17]. These results may be inconsistent because PAP is influenced by muscle fatigue. PAP is induced by a voluntary conditioning contraction; however, the voluntary conditioning contraction might also induce fatigue, and the balance between PAP and fatigue will determine the effect on the performance of explosive activity [18]. Research has shown that it is likely that the balance between fatigue and potentiation is more favorable with increased training experience [19]. On the other hand, the improvements in performance likely dissipate by 30 minutes after a conditioning stimulus [15]. Therefore, training experience and appropriate conditioning stimulus can help balance the fatigue-PAP relationship and induce the PAP effect to increase muscle power and improve athletic performance.

Acupuncture has time-dependent characteristics, and it has an immediate effect, and there is a cumulative effect of multiple acupuncture treatments [20]. Studies related to the immediate nature of acupuncture have shown that pain is relieved immediately after treatment in patients with pain symptoms [21]. For example, acupuncture therapy has an immediate effect on both motion-related pain and pain at rest in patients with cervical spine mobility disorders [22]. It also has an immediate effect on the mobility of patients with chronic neck pain [23]. Thus, acupuncture has an immediate effect and is used for the auxiliary treatment of pain. In a recent study on the immediate effect of acupuncture on performance in the drop jump task, acupuncture reduced the impact force during landing and may be used to reduce the risk of sports injury at a drop jump height of 50 cm (DJH50) [24]. Therefore, acupuncture is an alternative therapy that has been widely used in sports medicine to prevent sports injuries and improve sports performance. The cumulative effects are also commonly demonstrated in the treatment of clinical diseases; for example, patients with subacute hemorrhagic stroke showed the recovery of lower limb motor function after receiving 4 weeks of acupuncture treatment [25]. Patients with chronic knee pain (CKP) also showed effective pain relief after 12 weeks of acupuncture treatment [26]. A past study showed that acupuncture with Zhongwan (CV-12) for 30 minutes can significantly reduce the random blood glucose (RBG) level [27]. Therefore, multiple sessions of acupuncture have been used in the treatment of subacute and chronic diseases.

Although a recent study indicated that acupuncture can effectively increase the explosive strength of the shoulder joint [28], research on the time dependence of acupuncture effects to improve the explosive force of the shoulder muscles is still limited. Moreover, the efficiency of acupuncture may have clinical significance and lead to methodological advantages. Therefore, the purpose of the current study was to explore the effect of acupuncture on the timeliness of explosive forces generated by the male shoulder joint. The research hypothesis was that the impact of acupuncture on the explosive forces of the shoulder joint is time-dependent, and training programs to improve the explosive forces of the upper limbs of athletes can be formulated according to the time-related characteristics of acupuncture.

## 2. Materials and Methods

### 2.1. Study Design and Setting

We conducted a single-group pre- and posttest experiment at the Jilin Sport University Biomechanics Laboratory of the Health Technology College in Changchun, China. The experiments were conducted after receiving written consent from the subjects following a full explanation of the protocols. We registered the study in the Chinese Clinical Trial Registry (registration number: ChiCTR1900025407). The Ethics Committee of the Joint Institutional Review Board of Jilin Sport University (JLSU; Changchun, China; JLSU-IRB no. 2018004) approved this study.

### 2.2. Participants

Eighteen healthy male students (nonathletes; right-handed; age, 22.3 ± 1.2 years; height, 175.4 ± 5.8 cm; body mass, 63.2 ± 4.3 kg) were recruited through a recruitment announcement. They all volunteered to participate in this research at Jilin Sport University. Subjects were excluded from the study if they sustained musculoskeletal injuries or upper limb injuries within the past 6 months. The participants who drank beverages containing caffeine or alcohol within a period of less than 12 h prior to the measurements were also excluded [29]. All subjects had not received acupuncture or any medication in the last 6 months and were instructed to avoid all forms of exercise during the experiment.

### 2.3. Instruments

In this study, the shoulder joint muscle explosive forces were evaluated by using an isokinetic training system (Con-Trex MJ; CMV AG, Dübendorf, Switzerland). Disposable Hwato acupuncture needles (Suzhou Medical Appliance Factory, Suzhou, People's Republic of China) measuring 0.25 mm in diameter and 25 mm in length were used.

### 2.4. Acupuncture

**Figure 1:**
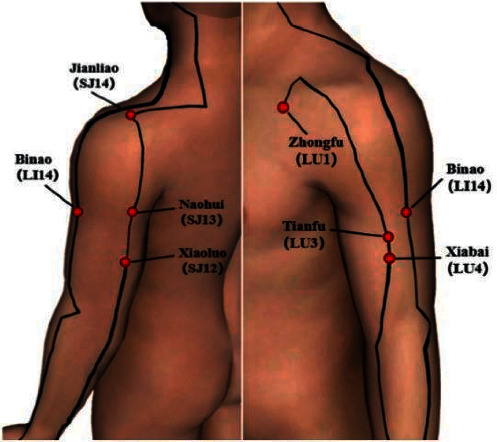
Acupuncture points.

Seven acupoints were selected according to the meridian system theory: two in the shoulder region (LU1 and SJ14) and five in the upper limbs (LU3, LU4, LI14, SJ13, and SJ12) (see Figure 1). A recent study has shown that acupuncture which was used to stimulate the LU1, LI3, LU4, LI14, SJ13, SJ14, and SJ12 points can effectively improve explosive force production of shoulder joint [28]. Therefore, in this study, we choose the same seven acupoints to explore the effect of acupuncture on the timeliness of explosive forces generated by the shoulder joint. And these points were chosen because they act as proximal points for joint movement, and acupuncture at these points can activate proximal muscles [30]. Under aseptic conditions, an experienced acupuncturist (JW) possess a Health Professional Qualification certificate, which is approved and issued by the Ministry of Human Resources and Social Security of the People's Republic of China and the National Health Commission, performed using disposable Hwato acupuncture needles (Suzhou Medical Appliance Factory, Suzhou, People's Republic of China) measuring 0.25 mm in diameter and 25 mm in length. And needles were carried out at a 90-degree angle to puncture the galea aponeurotica or skin. Following insertion, needles were held by the right thumb, forefinger, and middle finger and were manipulated by twirling, lifting, and thrusting to generate “De-qi”. The patients were asked if they feel “a sensation of soreness, numbness, heaviness, or distension around the point, or even a sensation travelling to a certain place” [31]. The needle was rotated at 2 min, 5 min, and 10 min after insertion, which is the needle rotating, lifting, and thrusting the needle gently with small amplitude for 20 min.

### 2.5. Protocol

**Figure 2:**
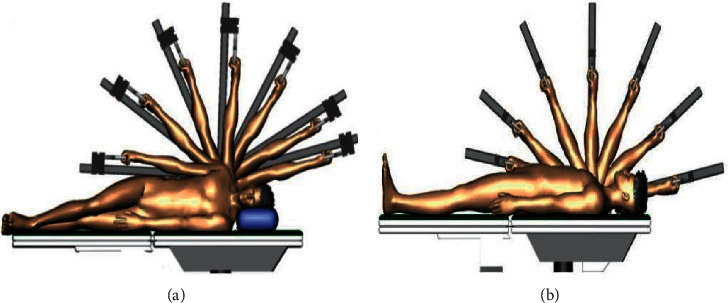
Schematic diagram of the trajectory and range of shoulder joint activity. (a) Shoulder joint adduction/abduction (Add/Abd) and (b) shoulder joint flexion/extension (Flex/Ext) movements.

**Figure 3:**
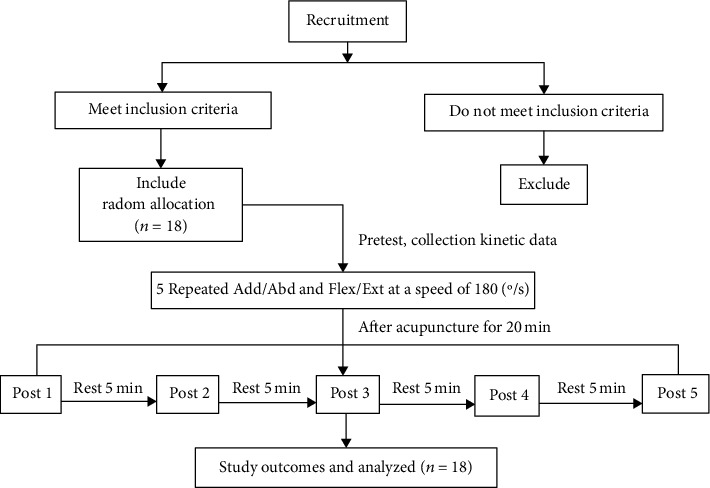
Flow diagram of the protocol for the study.

Prior to the trial, the subjects were informed about the study (the use of nonpenetrating needles; the possible risks, such as hematoma, and fainting; the procedure; and the purpose of the experiment) and asked to provide written informed consent. Participation in this experiment was voluntary; they could withdraw from the experiment at any time. The following experimental procedures were performed. (1) In the formal experiment, the subjects warmed up on a treadmill at a speed of 8 km/h for 5 minutes. (2) The participant lay comfortably with the upper limb unclothed. The right limb of the subject was used as the dominant limb to measure the values during constant velocity movement. The initial position of the subject was as follows: the shoulders were extended at 90°, the forearm was in a neutral position, and the shoulder was positioned at 90° in the horizontal plane. The axis of rotation of the device was aligned with the anatomical axis of the shoulder. (3) Each subject was required to complete five shoulder isokinetic muscle strength tests at a constant speed of 180 /s, which were recorded as the pretest. For each contraction, the participants were instructed to relax, take a deep breath, and following an auditory signal, contract their shoulder joint as “fast and hard” as possible for 1–1.5 s, with an emphasis on “fast.” The explosive contractions were performed until 15 contractions meeting these criteria were completed. The maximal voluntary force was defined as the largest force during any of the MVCs or explosive contractions [32]. The participants received verbal encouragement during the contractions, including shoulder joint Add/Abd (see Figure 2(a)) and Flex/Ext (see Figure 2(b)). All subjects did not have limited shoulder joint mobility in the entire range of motion. (4) The skin was cleaned with alcohol at the points to receive the needles. Then, the subjects received acupuncture stimulation at LU1, LI3, LU4, LI14, SJ13, SJ14, and SJ12 for 20 minutes. Stimulation was then delivered by a balanced “tonifying and reducing” technique, and the needles were rotated manually clockwise and counterclockwise for 1 min at a rate of 60 times per minute. (5) After acupuncture, the subjects rested for 5 minutes and performed full shoulder joint Abd/Add and Flex/Ext movements 15 times with an angular velocity of 180 /s, and the kinematic data were recorded as the data for post 1. After completing the assessment for post 1, the subjects rested for 5 minutes and then followed the same procedure for post 2. In total, each subject completed full shoulder joint Abd/Add and Flex/Ext movements six times (pre, post 1, post 2, post 3, post 4, and post 5) at different intervals (before acupuncture, 5 min, 13 min, 21 min, 29 min, and 37 min after acupuncture). The time to complete the isokinetic test is about 3 minutes, and the next isokinetic test is performed after a 5-minute rest and the interval is 8 minutes. Therefore, post 1 was performed 5 minutes after acupuncture and post 2 was performed 13 minutes after acupuncture. Similarly, post 3, post 4, and post 5 were 21 minutes, 29 minutes, and 37 minutes after acupuncture, respectively. All participants were asked to report any discomfort and ask questions during the experiment and received two sessions of isokinetic measurement before and after the acupuncture intervention. A flow diagram of the protocol is shown in Figure 3.

### 2.6. Data and Statistical Analysis

One-way analysis of variance (ANOVA) was used to analyze dependent variables among different time conditions. When significant differences were observed, Fisher's least significant difference (LSD) post hoc test was used to investigate relevant significant interactions between variables across time. And MATLAB (version R2016a; MathWorks, Inc., Natick, MA) also was used to analyze the raw data, and the isokinetic parameters including the max torque (Nm), the average max torque/kg (Nm/kg), the average work/kg (J/kg), the average power/kg (W/kg), the average peak power/kg (W/kg), and the total work (J) of the shoulder joint Add/Abd and Flex/Ext were recorded. The objective documentation that isokinetic provides in the examination, evaluation, diagnosis, prognosis, treatment interventions, and outcomes is particularly important for returning the patients safely and rapidly back to normal daily activities [33]. The maximum torque is the most widely used parameter to evaluate muscle function and is an excellent indicator of the subject's maximum strength level, and the maximum torque is the highest torque achieved during a series of repetitions. Total work is the total muscular force output for the repetition with the greatest amount of work. It is indicative of a muscle's capability to produce force throughout the range of motion [34]. Isokinetic motion improves muscle contractions by stimulating increased protein synthesis by muscle cells to produce more actin and myosin so that both the size and capacity of the muscle are increased to produce power output [35]. Past studies have shown that torque, work, power, and speed can be used as parameters for measuring explosive force [36]. Research shows that acupuncture can improve shoulder muscle endurance and explosive power, and the average maximum work, the average maximum power, the average maximum speed, and the total work were collected through an isokinetic test system to explore the difference before and after acupuncture [11, 28]. The significance level was set at *P* < 0.05, and data are presented as the mean ± SD, percent change (Δ%), and effect size (ES). Percent change was calculated as Δ% = ((post−pre/pre) *∗*100%), and predesignated ES range limits were established as follows: low effect = 0.20–0.49; medium effect = 0.50–0.79; large effect = 0.80–1.0.

## 3. Results

**Figure 4:**
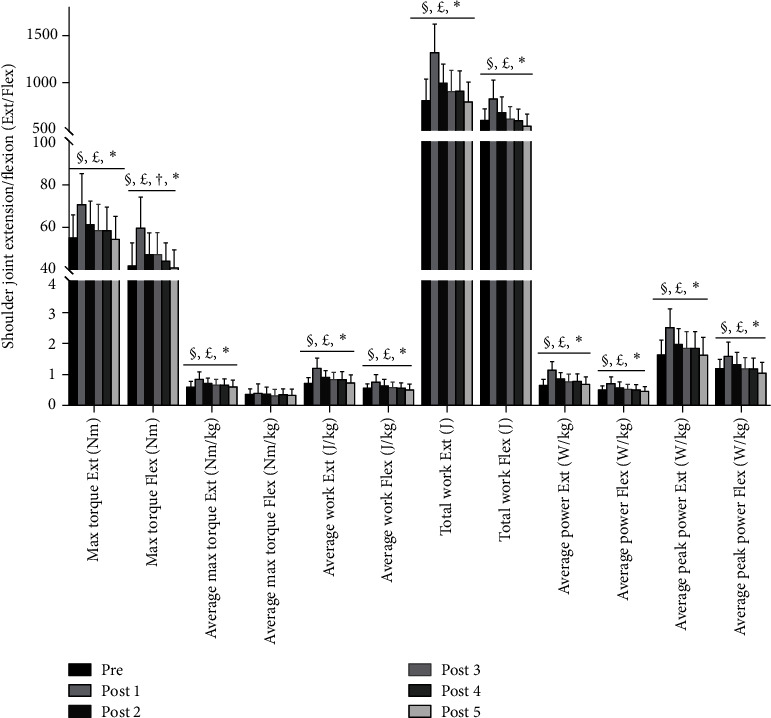
Isokinetic muscle force parameters and post hoc test differences for the shoulder joint in flexion/extension (Flex/Ext) before and after acupuncture. Note: values are mean ± SD. ^*∗*^Statistically significant predictor of shoulder joint Flex/Ext muscle explosive force (*P* < 0.05). ^§^A significant difference from the pretest to post 1 (*P* < 0.05). ^£^A significant difference from the pretest to post 2 (*P* < 0.05). ^†^A significant difference from the pretest to post 3 (*P* < 0.05).

All participants completed the study procedure with no adverse reactions reported. The ANOVA test revealed a significant main effect of time on the shoulder joint Flex/Ext torque, work, and power before and after acupuncture (*P* < 0.05). The LSD post hoc tests indicated that the max torque Flex/Ext, respectively, significantly increased in both post 1 and post 2 compared with pre (post 1: +△43%, ES = 1.228, +△29%, ES = 1.211; post 2: +△11%, ES = 0.774, +△13%, ES = 0.621; all *P* < 0.05); the average max torque Ext in post 1 and post 2, respectively, significantly increased (+△41%, ES = 1.419; +△16%; ES = 1.096; all *P* < 0.05); the average work Flex/Ext, respectively, significantly increased (post 1: +△38%, ES = 1.165, +△65%, ES = 1.538; post 2: +△14%, ES = 0.647, +△25%, ES = 0.802; all *P* < 0.05); the average power Flex/Ext, respectively, significantly increased (post 1: +△44%, ES = 1.270, +△72%, ES = 1.578; post 2: +△17%, ES = 0.709, +^△^29%, ES = 0.860; all *P* < 0.05); the average peak power Ext/Flex, respectively, significantly increased (post 1: +△35%, ES = 1.312, +△53%, ES = 1.597; post 2: +△12%, ES = 0.616, +△21%, ES = 0.786; all *P* < 0.05); and the total work Ext/Flex, respectively, significantly increased (post 1: +△37, ES = 1.226, +△62%, ES = 1.661; post 2: +△13%, ES = 0.575, +△23%, ES = 0.738; all *P* < 0.05). Additionally, the post 3 value of max torque Flex significant increased compared with the pre value (+△13%, ES = 0.596, *P* < 0.05). No differences were found in average max torque Flex (*P* < 0.05) across the study, and therefore, it was removed from the post hoc tests. Isokinetic muscle force parameters and post hoc test differences for the shoulder joint in Flex/Ext are presented in Figure 4.

Similarly, the ANOVA test also revealed a significant main effect of time on the shoulder joint Add/Abd torque, work, and power before and after acupuncture (*P* < 0.05). The LSD post hoc tests indicated that the max torque Add/Abd, respectively, significantly increased in both post 1 and post 2 compared with pre (post 1: +△23%, ES = 1.675, +△32%, ES = 1.524; post 2: +△11%, ES = 0.555, +△16%, ES = 0.552; all *P* < 0.05); the average max torque Add in post 1 and post 2, respectively, significantly increased (post 1: +△33%, ES = 2.112; post 2: +△22%; ES = 0.968; all *P* < 0.05); the average work Add/Abd, respectively, significantly increased (post 1: +△46%, ES = 2.020, +△27%, ES = 1.560; post 2: +△34%, ES = 0.916, +△23%, ES = 0.863; all *P* < 0.05); the average power Add/Abd, respectively, significantly increased (post 1: +△63%, ES = 2.427, +△48%, ES = 2.215; post 2: +△40%, ES = 1.317, +△35%, ES = 1.129; post 3: +△27%, ES = 0.771, +△19%, ES = 0.635; all *P* < 0.05); the average peak power Add/Abd, respectively, significantly increased (post 1: +△46%, ES = 2.576, +△42%, ES = 1.915; post 2: +△29%, ES = 1.043,+△29%, ES = 0.950; all *P* < 0.05); and the total work Add/Abd, respectively, significantly increased (post 1: +△45%, ES = 1.986, +△33%, ES = 1.698; post 2: +△26%, ES = 0.915, +△21%, ES = 0.866; all *P* < 0.05).

**Figure 5:**
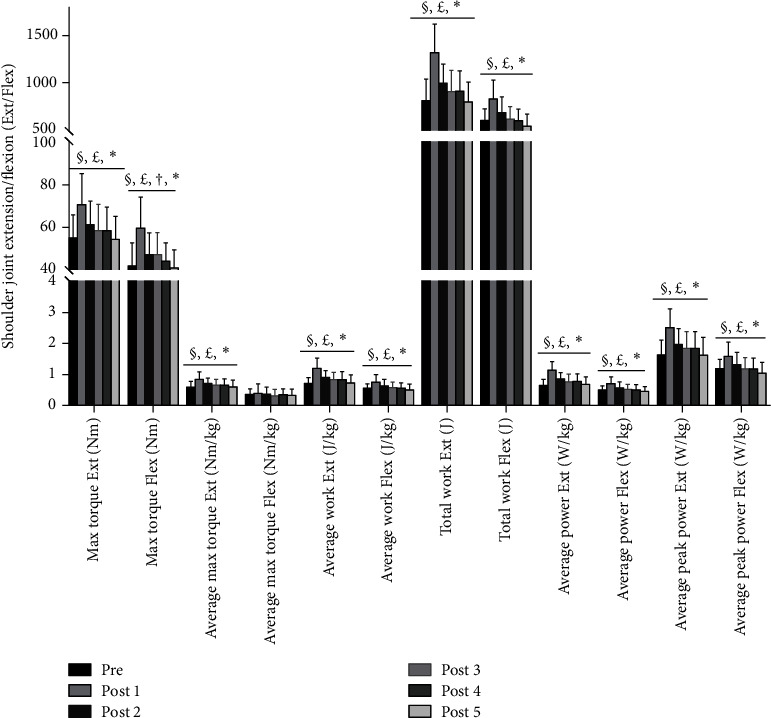
Isokinetic muscle force parameters and post hoc test differences for the shoulder joint in adduction/abduction (Add/Abd) before and after acupuncture. Note: values are mean ± SD. ^*∗*^Statistically significant predictor of shoulder joint Add/Abd muscle explosive force (*P* < 0.05). ^§^A significant difference from the pretest to post 1 (*P* < 0.05). ^£^A significant difference from the pretest to post 2 (*P* < 0.05). ^†^: indicates a significant difference from the pretest to post 3 (*P* < 0.05).

Additionally, post 3 value of average work Add (+△17%, ES = 0.551, *P* < 0.05) and the average peak power Add (+△20%, ES = 0.631, *P* < 0.05) significantly increased compared with the pre value. No differences were found in average max torque Abd across the study, and therefore, it was removed from the post hoc tests (*P* < 0.05). Isokinetic muscle force parameters and post hoc test differences for the shoulder joint in Add/Abd are presented in Figure 5.

## 4. Discussion

This study assessed the effects of acupuncture on the timeliness of explosive forces generated by the male shoulder joint. The results of this study show that acupuncture can increase the torque, work, and power of the shoulder joint muscle groups. Therefore, it can improve the muscle explosive forces of the male shoulder joint and the effect takes approximately 10 minutes to appear.

### 4.1. Dynamic Data Analysis of the Shoulder Joint in Flex/Ext before Acupuncture (Pre) and after Acupuncture (Post 1 and Post 2)

The pre value of shoulder joint Flex/Ext torque value (max torque and average max torque), work (average work and total work), and power (average power and average peak power) decreased compared with post 1 after acupuncture, except for the average max torque Flex. A previous study showed that acupuncture is able to increase carnitine levels and thus decrease the level of fatigue of skeletal muscle [37]. When skeletal muscle contracts, the flow of Ca^2+^ to the sarcoplasmic reticulum increases greatly, and the sensitivity of the binding site to Ca^2+^ increases. And the total amount of activated myosin adenosine triphosphate (ATPase) also increases, and the rate of ATP release energy increases, which improves the muscle globulin and actin cross-bridge swing rate, thereby enhancing the power of skeletal muscle contractions [38]. Therefore, in this study, acupuncture increases the work and power of the shoulder joint by inducing neurophysiological responses in the body to increase the contractions of skeletal muscles.

### 4.2. Dynamic Data Analysis of the Shoulder Joint in Add/Abd before Acupuncture (Pre) and after Acupuncture (Post 1 and Post 2)

The pre value of shoulder joint Add/Abd torque value (max torque and average max torque), work (average work and total work), and power (average power and average peak power) of the shoulder joint Add/Abd decreased compared with post 1 after acupuncture, except for the average max torque Abd. Previous studies have shown that acupuncture stimulates nerves, increasing the recruitment of motor units and increasing muscle activity [6], and stimulating acupuncture points can also improve biomechanical indexes, including the maximum peak force moment, acceleration of force, and average power, thereby improving athletes' athletic performance [39]. Therefore, in this study, the Add/Abd values of the shoulder joint increased after acupuncture at post 1, including the torque, work, and power values, which may be due to the increase in the biomechanical index and body function of the shoulder joint by the acupuncture nerve, thereby increasing the explosive force of the shoulder joint.

However, the average max torque values in Abd and Flex did not differ before and after acupuncture. Acupuncture may cause the activation of peripheral muscle receptors and increase the number of activation receptors, leading to an increase in the central nervous system excitability and the physiological response of “De-qi” in the muscles and peripheral motor neurons at the acupuncture site [30]. This response may increase muscle strength by stimulating nerves [11]. However, the study found no correlation between direct stimulation of peripheral nerves and “De-qi” sensation [40]. And in some cases, even if a needle is inserted into a nerve, “De-qi” cannot be induced [40]. This has been shown in past studies that “De-qi” sensation was well achieved before the needle touched the median nerve under neiguan acupoint, suggesting that irritation of the nerve was not directly involved in generating it [41]. Therefore, in this study, acupuncture at points Lu1, Lu3, Lu4, SJ4, and Li4 failed to increase the average maximum torque of shoulder abduction/flexion before and after acupuncture, which may be because acupuncture at these points cannot cause the production of “De-qi” sensation, so that the torque value tested in the repeated action failed to increase.

### 4.3. Timeliness Analysis of the Shoulder Joint in Add/Abd and Flex/Ext before and after Acupuncture

There were significant differences in the torque, work, and power of the shoulder joint in Add/Abd and Flex/Ext between pretest to post 2, but there were no differences between pretest to post 3, post 4, and post 5. This finding shows that acupuncture takes approximately 10 minutes to increase the explosive forces of the shoulder joint in Add/Abd and Flex/Ext. A previous study showed that a natural increase in the neural activity in response to an acupuncture stimulus may remain elevated for a period of time and not return to the prestimulation baseline level during subsequent interstimulus epochs of rest, which would confound the estimate of how much the neural activity increases/decreases during subsequent epochs of stimulation [42]. Another study showed that acupuncture has an immediate effect on pain in the neck and shoulder regions, but the effect did not last until the next treatment [43]. Acupuncture at Hegu point can induce a decrease in the motor evoked potential of the abductor digitorum, and the effect still exists within 15 minutes after the acupuncture is removed [44]. Accordingly, in this study, the effect of acupuncture on the explosive forces in Add/Abd and Flex/Ext of the shoulder joint decreased over time, and the effect took approximately 10 minutes to appear. In the future design of experimental trials involving acupuncture, we will add to the discussion how to incorporate acupuncture into the training plan based on the current research results to provide more clinical significance for the research.

## 5. Limitations

Although the results are useful, there are several limitations of the present study that warrant some discussion. First, this study is subjective in terms of “De-qi.” Namely, we asked patients to self-report their sensory experiences rather than using objective measures, such as the acupoint surface temperature and evoked somatosensory potentials, to evaluate “De-qi.” We did not measure muscle blood flow to study the mechanism of acupuncture. Another limitation is that a sham acupuncture intervention was not included. Only male subjects were recruited to participate in this study, so the gender differences on shoulder joint muscle explosive force will be further studied and discussed in the following research. In addition, the experiment (isokinetic test) of this study was completed in the laboratory, which may be different from the actual field test (e.g., pitching and throwing) results, and we will further improve the translation between lab assessment and field test.

## 6. Conclusions

Our research results show that acupuncture at specific points on the shoulder join Add/Abd and Flex/Ext muscles for 20 minutes can effectively improve muscle force. Moreover, the post 1 and post 2 values after acupuncture are compared with the pre before acupuncture, and it was found that the timeliness of improving shoulder joint explosive force is about 10 minutes. In conclusion, this study facilitates a better understanding of acupuncture stimulation which can improve muscle explosive force and its time effects. It is recommended to extend this research to athletes, especially those who want to improve their explosive force, athletic performance, and competition score in a short period.

## Data Availability

The datasets used and analyzed in the current study are included in this article.
